# A Series of Anatomical Sketches and Diagrams

**Published:** 1841-10

**Authors:** 


Art. XIV.—*A Series of Anatomical Sketches and Diagrams.* By Thomas Wormald, and A. M. M'Whinnie, of St. Bartholomew's Hospital.—*London*, 1841. Part IV.

The present number of this elegant and useful series of Anatomical Sketches contains views of the subclavian, thyroid, and axillary regions, as well as two drawings of the superficial and deep relation of parts at the bend of the elbow, and a diagram of the communication between the spinal nerves and sympathetic ganglia. They are equally unpretending and bear the same impress of accuracy as those of the numbers which preceded them. They need not our recommendation, for they cannot fail to recommend themselves to the anatomical student, who desires just so much aid of this nature as he ought to receive in the prosecution of his dissections.

